# Income Level and Survival After Resection of Liver Metastases: A Population‐Based Study

**DOI:** 10.1002/wjs.70036

**Published:** 2025-08-08

**Authors:** Cecilia Strömberg, Mirna Abraham‐Nordling

**Affiliations:** ^1^ Division of Surgery Department of Clinical Science Intervention and Technology (CLINTEC) Karolinska Institutet and Karolinska University Hospital Stockholm Sweden; ^2^ Department of Molecular Medicine and Surgery Karolinska Institutet Stockholm Sweden; ^3^ Department of Pelvic Cancer Division of Coloproctology Center for Digestive Diseases Karolinska University Hospital Stockholm Sweden

**Keywords:** colorectal cancer, income level, liver metastases, socioeconomic factors

## Abstract

**Background:**

This study aimed to investigate whether socioeconomic factors are associated with an improved long‐term survival after colorectal cancer liver metastases (LM) resection.

**Methods:**

A retrospective nationwide population‐based cohort study. All patients who underwent hepatectomy in Sweden between 2002 and 2011 were identified in the Swedish Hospital Discharge Registry using their unique personal identification numbers. This cohort was linked to the National Cancer Registry (cancer diagnosis), the National Registry of Causes of Death, and the Migration Registry. Survival analysis by the Kaplan–Meier method was performed to assess long‐term outcome. A Cox regression model was used to analyze risk factors affecting long‐term survival.

**Results:**

In total, 2874 liver resections were performed in 2327 patients who were included in the study. In the group, 1362 (59%) were male and the median age was 65 (3–89) years. Patients that had more than one resection, the time was counted from the last resection. The main resection was a local resection that was performed in 60% of patients. The overall 5‐year survival rate was 48%. In the univariate analysis, there was a significant association in overall survival for age, comorbidity, sex, income, and education level. In the adjusted multivariate logistic regression analysis, there was a significant relation with (higher OS, for lower age (HR 1.46 (95% CI: 1.23–1.72) and *p* < 0.001), lower comorbidity (HR 0.78 (95% CI: 0.66–0.92) and *p* < 0.003), female sex (HR 1.24 (95% CI: 1.05–1.47) and *p* = 0.01), and higher income (HR 0.84 (95% CI: 0.71–0.99) and *p* = 0.045)).

**Conclusions:**

Patients that underwent surgery for LM from mainly colorectal disease and who have a low‐income experience shorter overall survival. Further findings showed that patients with lower age and comorbidity and female sex had longer overall survival.

## Graphical abstract

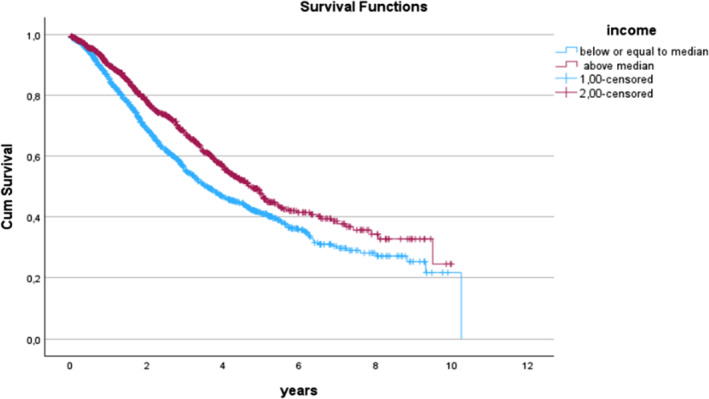

## 1 Introduction

Colorectal cancer (CRC) ranks as the third most common cancer worldwide, and approximately 15%–30% of patients present with metastases [1]. The liver is one of the most common locations of distant colorectal metastases [2, 3] and liver resection as well as ablation was during the time period considered the only potentially curative treatment for such metastases. However, due to the size or number of lesions and/or the location of extrahepatic disease, no more than around 20% of patients with liver metastases (LM) are suitable candidates for liver resection [4]. The five‐year overall survival (OS) after liver resection ranges from 30% to 50%, whereas disease‐free survival (DFS) ranges from 25% to 35% [4, 5, 6].

Most studies on the prognosis and long‐term mortality following liver surgery are based on highly specialized or single institution cohorts [7, 8]. Additionally, there are limited population‐based studies that report the effects of liver surgery on a general population, including the impact on prognosis and long‐term mortality after colorectal liver metastatic surgery. Several studies have reported that a patient's access to high‐volume and highly specialized centers predict a better prognosis and mortality rate [9, 10, 11, 12]. This supports the theory and previous reports that surgeons training and skill level has impact on the incidence of postoperative complications [13]. This highlights the challenge faced by healthcare systems to provide equitable healthcare to all citizens regardless of their home district.

There is a lack of studies assessing whether factors, such as home district, sex, social status, education level, and income, affect the prognosis and long‐term survival after following colorectal liver metastases (CRLM) resection. Nevertheless, data indicate a favorable long‐term prognosis after cardiovascular and esophageal surgery for married patients and those with a higher education level [14, 15, 16, 17, 18, 19, 20, 21, 22].

The aim of the current study was to determine whether socioeconomic factors of patients impact long‐term survival as a means of evaluating the provision of equal healthcare in Sweden.

## 2 Material and Methods

### 2.1 Study Population

All patients that have underwent hepatectomy due liver metastases, mainly from colorectal cancer, in Sweden during the period 2002–2011 were included in the study cohort.

The data were collected from four separate registries that were interlinked through the patients' national identification number. The registries are endorsed and supervised by the Swedish Board of Health and Welfare and Statistics Sweden (SCB).

The Swedish Hospital Discharge Registry was founded in 1965 and is complete since 1987, it includes all in‐hospital patient contacts; these can be traced through the patients' registration number for identification. In this study, all patients were identified by their unique national registration numbers in the Hospital Discharge Register while matching an in‐hospital discharge procedure code for liver resection according to the 10th revision of the ICD 10 (International Classification of Diseases and procedures, codes JJB00, JJB10, JJB20, JJB30, JJB40, JJB50, JJB53, JJB60, JJB71, and JJB96). Data on patients' characteristics as age, sex, diagnosis, and comorbidity as well as home district were obtained from the Hospital Discharge Registry. By cross‐linkage to Statistics Sweden using patient's national identification number, information of their civilian records as marital status, income, and education level were retrieved. Moreover, the personal national registration number was used for crosslinking with the Swedish Registry of Causes of Death and to the Registry of Domestic and International relocations to analyze postoperative and long‐term survival outcome. The cohort was followed until death, emigration, or end of follow‐up until December 31st, 2011.

The cohort study data were from 1st January 2002 to 31st December 2011. A detailed description of the methods used in this study has been described elsewhere [23].

### 2.2 Study Exposures

Information on income, social status, and education level was collected from the Swedish National education registry held by Statistics Sweden. The variable income was categorized into two levels, below or above median income in 2002 (219,233 Swedish krona (SEK)/year) not taken in consideration the inflation during the period, since that was low (0%–3%) according to Statistics Sweden.

Social status was categorized as married/cohabitating couples or single and in conformity with previous studies [19]. Single status also included patients who were divorced or widowed.

The registry is updated yearly and the highest education level during the study period for each subject was used. Education level was defined by Swedish Educational Nomenclature (SUN 2000) [24] in conformity with UNESCO's ISCED classification but applicable on the Swedish education system. Seven levels are used in the registry: (1) Early childhood education, (2) primary education shorter than nine years, (3) primary education nine or 10 years, (4) secondary education, (5) tertiary education shorter than 2 years, (6) tertiary education 2 years or longer, and (7) tertiary education on doctoral or equivalent level. Masters' degree did not exist in Sweden at the time. Education level was categorized in two levels: University level or not in accordance with previous reports [25].

In the risk factor analysis, Data on hospital liver resection volume were collected from the Hospital Discharge Registry. Hospitals were categorized into nonuniversity and university hospitals as well as high volume (≥ 300 resections during the study period) and low volume (< 300 resection during the 10‐year period). There was 7 university and 33 nonuniversity hospitals in the study.

Sweden is divided in six regions: (1) Uppsala and Örebro region (reference) contains two university hospitals, (2) Lund region, (3) Stockholm region, (4) Gothenburg region, (5) Umeå region, and (6) Linköping region. During 2002–2011, liver surgery was performed also outside university hospitals.

As potential confounders age, comorbidity, and extent of surgery were identified and included in the analyses to correct for bias. Age was categorized under or above the median > 65 years at the time for surgery. Comorbidity, classified according to the Charlson score (Charlson 1987) [26], was modified due to the fact that almost all patients had a diagnosis of malignancy, which was excluded from the score. The respective variables were categorized into two groups: Charlson score 0–1 or ≥ 2.

The diagnosis that was stated in the discharge record was used as a surrogate for the indication for surgery. Accordingly, we identified the following diagnoses: metastases (ICD10 code C78.7). The cohort was further defined by adding the inclusion criteria of a diagnosis of colorectal cancer (ICD10‐codes C18.0‐C18.9, C19.9, and C20.9).

### 2.3 Ethical Approval

The study was approved by the local Ethical Committee of the Karolinska Institutet (2010/1872–31/2).

### 2.4 Statistical Analysis

Data were calculated as median (range) for continuous variables and proportions for categorical variables. Long‐term survival after liver‐resection was assessed by the Kaplan–Meier method.

Cox proportional hazard ratios (HRs) with 95% confidence intervals (CIs) were used for univariable and multivariable assessments of the association between potential risk factors and the hazard, that is, risk of death of all causes with time‐at‐risk as the underlying time‐scale. Potential risk factors used in the regression modeling were categorized in order to facilitate the analyses. By introducing the variables stepwise into the multivariable regression model tested potential confounding effects, and the risk factors were also tested for possible statistical interactions. A *p*‐value less or equal 0.05 was considered to be statistically significant. Statistical analyses were performed using SPSS version 20 for Windows (SPSS Inc., Chicago, IL).

## 3 Result

In total 2327 patients were included in the final analysis, 1362 (59% male) patients and the median age was 65 years, Table 1. The local resection (atypical resection, nonanatomical segment resection, or not fully liver segment) was the main resection that was performed in 60% of the patients. The Charlson score was mainly Charlson 0 (66%) or Charlson 1 (33%). The median income for the whole group was 219,233 SEK (range 0–4,161,088 SEK). The two common education level among the patients were primary education nine or ten years *n* = 487 (21%) and early childhood education *n* = 333 (14%). A majority of the patients were married/cohabitating couples (58%), Table 1. Patients who had one or several reresections were included from the last resection to avoid immortality bias.

**TABLE 1:** Patient characteristics for liver metastases.

	Patients treated with liver resection for liver metastases *n* = 2327
Age at time for surgery, median years (range)	65 (3–89)
Sex, male (%)	1362 (58.5)
Type of liver resection *n* (%)	
Local resection	1398 (60.1)
Hemihepatectomy	767 (33.0)
Extended hemihepatectomy	162 (7.0)
Re‐resection, *n* (%)	532 (18.5)
Comorbidity (Charlson excluding malignancy) *n*, (%)	
0	1542 (66.3)
1	764 (32.8)
2	9 (0.4)
> 2	12 (0.5)
Income, median (range) SEK	219,233 (0 – 4,161,088)
Education *n* (%)	
1 Early childhood education	333 (14.3)
2 primary education shorter than nine years	162 (7.0)
3 primary education nine or 10 years	487 (20.9)
4 secondary education	263 (11.3)
5 tertiary education shorter than 2 years	202 (8.7)
6 tertiary education 2 years or longer	298 (12.8)
7 tertiary education on doctoral level	27 (1.2)
8 data missing	555 (23.9)
Marital status *n* (%)	
Married/cohabitating couples	1356 (58.3)
Single, divorced, or widowed	821 (35.3)
Data missing	150 (6.4)

Abbreviation: SEK, Swedish krona.

### 3.1 Overall Survival in the Group

**FIGURE 1:**
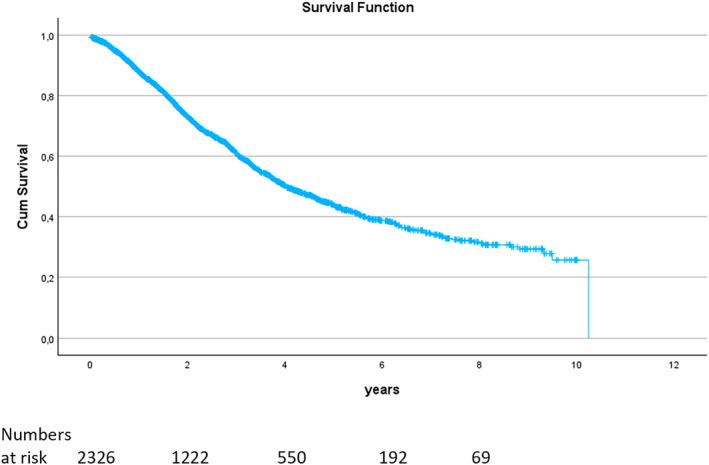
Kaplan–Meier survival estimates for all liver metastases resection in the cohort.

The 5‐year overall survival for the whole group was 48% and mean 4.58 years (95% CI 4.28–4.88), Figure 1.

### 3.2 Overall Survival and Risk Factors

Univariable and multivariable risk factor analyses for the long‐term outcome after LM resection is shown in detail in Table 2. When analyzing survival after surgery, it was revealed in the univariate analysis that there was a significant association in overall survival for age, comorbidity, sex, education level, and income. These parameters were included in the multivariate analysis.

**TABLE 2:** Univariable and multivariable (Cox) regression analyses of risk factors for mortality (long‐term survival).

	Univariate analysis		Multivariate analysis^a^	
	HR (95% CI)	*p*‐value	HR (95% CI)	*p*‐value
Age, > 65 years	1.45 (1.27–1.65)	< 0.001	1.46 (1.23–1.72)	< 0.001
Comorbidity, Charlson 0–1	0.76 (0.66–0.87)	< 0.001	0.78 (0.66–0.92)	0.003
Sex, male	1.15 (1.01–1.31)	0.029	1.24 (1.05–1.47)	0.01
Type of liver resection:				
Local resection	Ref			
Hemihepatectomy	0.85 (0.67–1.08)			
Extended	1.08 (0.85–1.39)			
Income above median	0.78 (0.66–0.86)	< 0.001	0.84 (0.71–0.99)	0.045
Education, tertiary	0.83 (0.70–0.99)	0.04	0.92 (0.77–1.11)	
Marital status, married/cohabitating couples	0.90 (0.77–1.04)			

Abbreviations: HR, hazard ratio and 95%, CI 95% confidence interval.

^a^ Adjusted for, age above or below median (65 years), Comorbidity Charlson (without malignancy) 0–1 or above, resection local, hemihepatectomy or extended, income below or above median (219,233 SEK), education university level or not, and marital status single/divorced/widowed or married/cohabitating couples.

**FIGURE 2:**
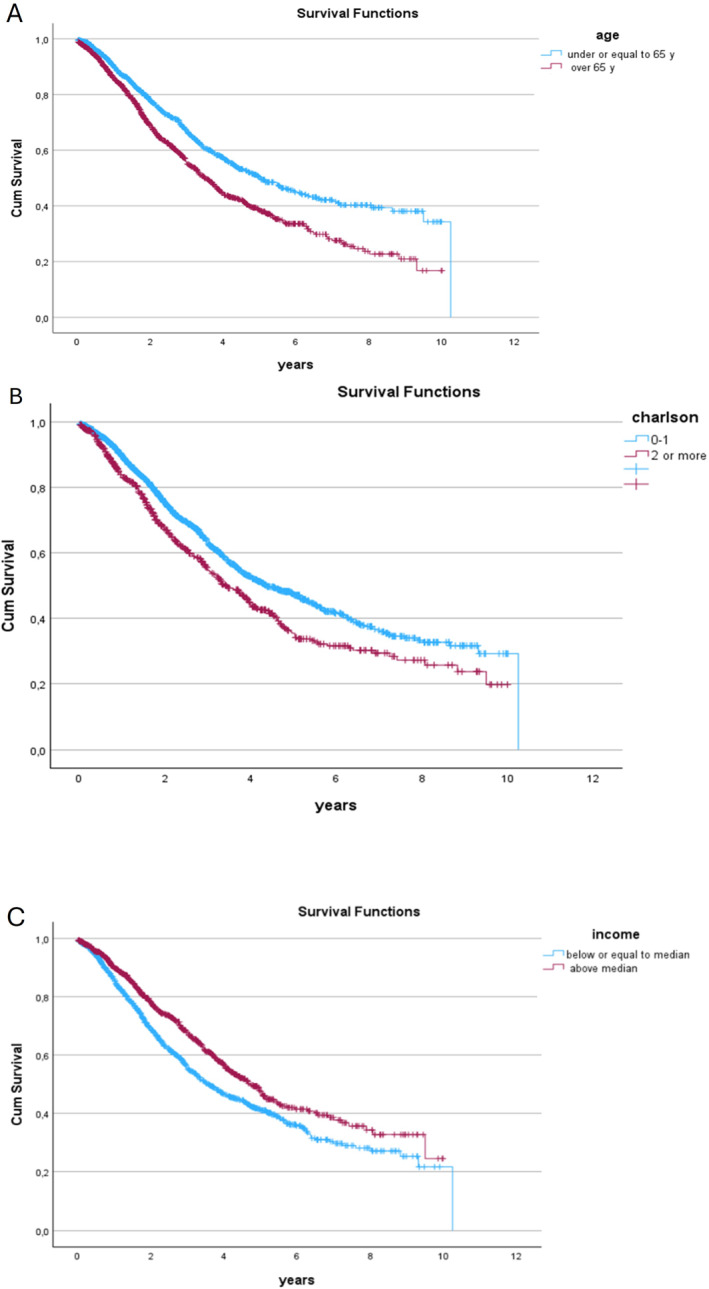
Kaplan–Meier survival estimates for patients with liver metastases resection (A) age, (B) comorbidity, and (C) income level.

Where we found a significant relation with higher OS with, lower age (HR 1.46 (95% CI: 1.23–1.72) and *p* < 0.001), lower comorbidity (HR 0.78 (95% CI: 0.66–0.92) and *p* < 0.003), female sex (HR 1.24 (95% CI: 1.05–1.47) and *p* = 0.01), and higher income (HR 0.84 (95% CI: 0.71–0.99) and *p* = 0.045). Education level did not reach the level of significance when we adjusted for the other factors Table 2. Figure 2. There were small regional differences in OS, in region 4, there was a significantly lower OS in the multivariate HR 1.33 (95% CI: 1.09–1.62) *p* = 0.006), when adjusted for age and Comorbidity.

## 4 Discussion

In this nationwide population‐based study of patients undergoing mainly colorectal liver metastatic (CRLM) resection in Sweden, we identified an association between patients with high disposable income and a higher overall survival. To our knowledge, this is the first study to correlate patient income with overall survival after liver resection related to metastasis from mainly colorectal cancer. A previous large study in Sweden by Noren et al. [27] did not find a correlation between income or education level and the odds of undergoing liver resection for CRLM. However, the main aim of that study was to investigate the selection process for liver surgery in patients with synchronous colorectal cancer metastases as well as reporting on survival in a nationwide setting.

The current study's five‐year OS of 48% was consistent with the previously published range of 30%–50% [4, 5, 6], suggesting that the study cohort and methodology are reliable.

Furthermore, the socioeconomic disparities in survival have also been reported from a national databases study from United Kingdom. This study reported that socioeconomic deprivation was associated with lower rates of liver resection and reduced 3‐year survival among patients suffering from CRC with synchronous liver‐limited metastases. Patients in the least deprived quintile exhibited better 3‐year survival compared to those in the most deprived quintile (least deprived vs. most deprived quintile, 22.3% vs. 17.4%; adjusted (HR 1.20 (95% CI: 1.11–1.30)) [28]. Additionally, a large study by Sell et al. confirmed these findings, demonstrating that patients with CRC and synchronous liver metastases with higher incomes were more likely to undergo liver resection, which was associated with improved overall survival [29].

In the current study, there was a significant association for low income and a lower education level with shorter survival time in the unadjusted analyses, but in the multivariate analysis, the association of lower education level on survival was not significant. Therefore, larger studies are needed to statistically verify the level of potential associations for educational level suggested by the present study. We anticipated that education level would influence patient outcomes. Yet, when education was categorized into university and nonuniversity levels, no significant impact was observed, which might support our hypothesis of equitable healthcare access in Sweden.

However, the income data revealed different insights. Patients with a median income above approximately 200,000 SEK per year exhibited better survival rates. The finding that no association exists between lower education and worse long‐term overall survival has been reported previously in studies on esophageal cancer surgery in Sweden [15]. This could indicate a threshold effect for both education and income levels below or above, which there is no significant impact on patient survival outcomes.

The study revealed a slight difference in OS for one the regions, which deserves further investigations, as this might be an indication of an inequal healthcare system.

Although Sweden provides universal healthcare through the National Health Service, the current study reveals noticeable health disparities between individuals with different income levels and to a lesser degree among the investigated regions.

There are several strengths of this study, including its nationwide and population‐based prospective cohort comprising 2327 patients. The results have been adjusted for confounding by known prognostic factors. Furthermore, the registries used for this study are reliable, as previous validation studies have reported a 95% accuracy concerning procedure codes in the Hospital Discharge Registry and nearly 100% registration in both the Registry of Causes for Death and National Cancer Registry [30].

However, there are also some limitations associated with the observational design and limited clinical variables provided by the registers. For instance, data on colorectal cancer stage was not available, which may impact the overall survival (OS) results. Another limitation is that patients were categorized into different education and income levels based on the year of diagnosis, without analyzing the development of these factors over time for individual patients. Furthermore, the analysis focused on individual patient income rather than household disposable income, potentially affecting OS if the income of other household members is significantly different. Finally, confounding by factors not adjusted for could influence the results.

## 5 Conclusion

The study indicates that patients who underwent surgery for mainly colorectal cancer (CRC) liver metastases and have a low‐income experience shorter overall survival. It is imperative to implement guidelines ensuring that patients with low socioeconomic status receive comprehensive information and support throughout their treatment and follow‐up care.

## Author Contributions

**Mirna Abraham Nordling:** conceptualization, data curation, investigation, validation, writing – original draft, writing – review and editing. **Cecilia Strömberg:** conceptualization, data curation, formal analysis, investigation, validation, writing – original draft, writing – review and editing.

## Consent

The authors have nothing to report.

## Conflicts of Interest

The authors declare no conflicts of interest.

## Data Availability

The data that support the findings of this study are available from the corresponding author upon reasonable request.
